# Supplementary material for: Evolutionary Shortcuts via Multinucleotide Substitutions and Their Impact on Natural Selection Analyses
Source: Mol Biol Evol. 2023 Jul 3;40(7):msad150. doi: 10.1093/molbev/msad150 (PMC10336034; doi:10.1093/molbev/msad150)
Supplement: msad150_Supplementary_Data [file msad150_supplementary_data.pdf]

· doi:10.1093/molbev/mst012 MBE

1 ## Supplementary Material

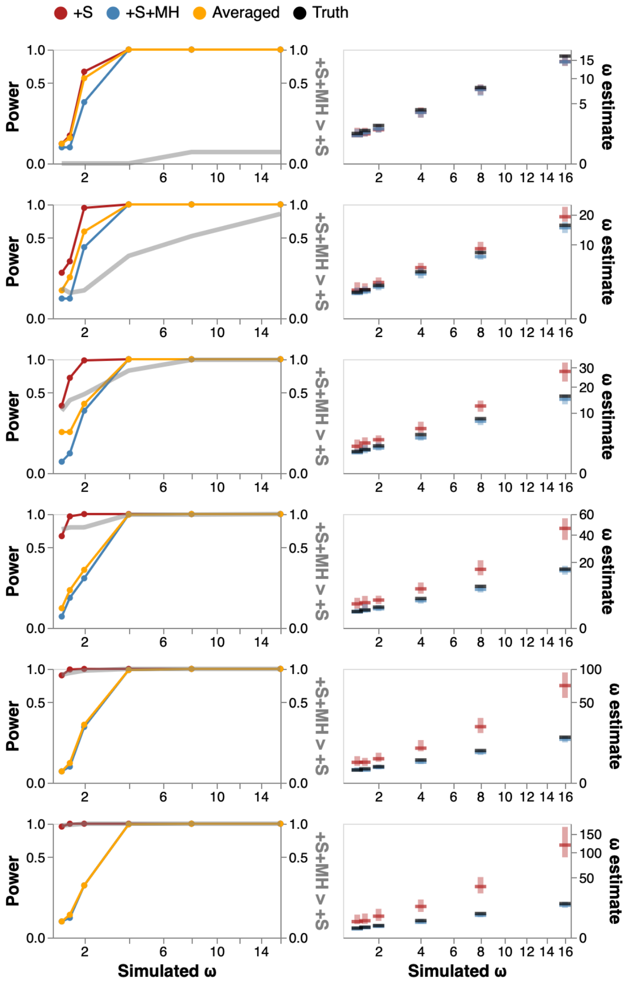

FIG. S1. Model performance on data simulated with EDS (25% selected fraction). Left column : detection rate for EDS (at $p \leq 0.05$) as a function of rate ω_3 (effect size) and δ (confounding parameter), and the rate at which +S+MH is preferred to +S by a nested LRT test. Right column: ω_3 estimates (median, IQR) for various simulation scenarios.

· doi:10.1093/molbev/mst012 MBE

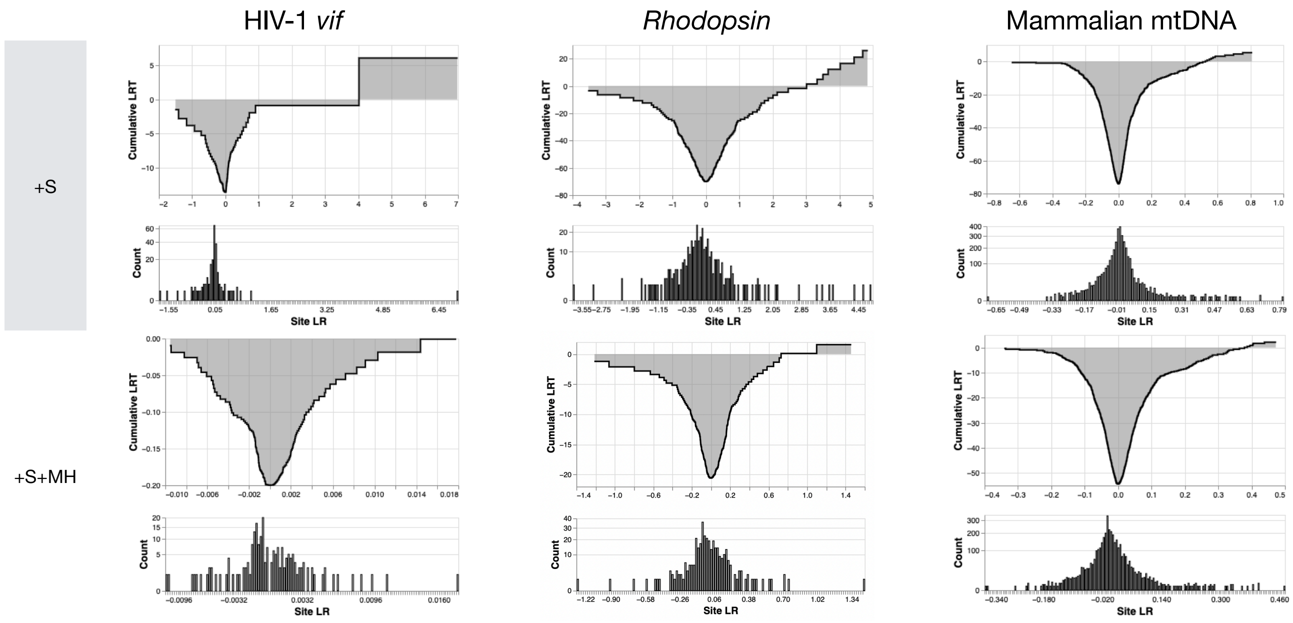

FIG. S2. Site-level support for Episodic Diversifying Selection in three benchmark alignments. Each dataset / model panel includes two views of the same data: the top plot is the cumulative value of the likelihood ratio test statistic (LRT) for the EDS test over sites, where site-level LRT are sorted from smallest to largest; the bottom plot is the histogram of site-level LRTs.

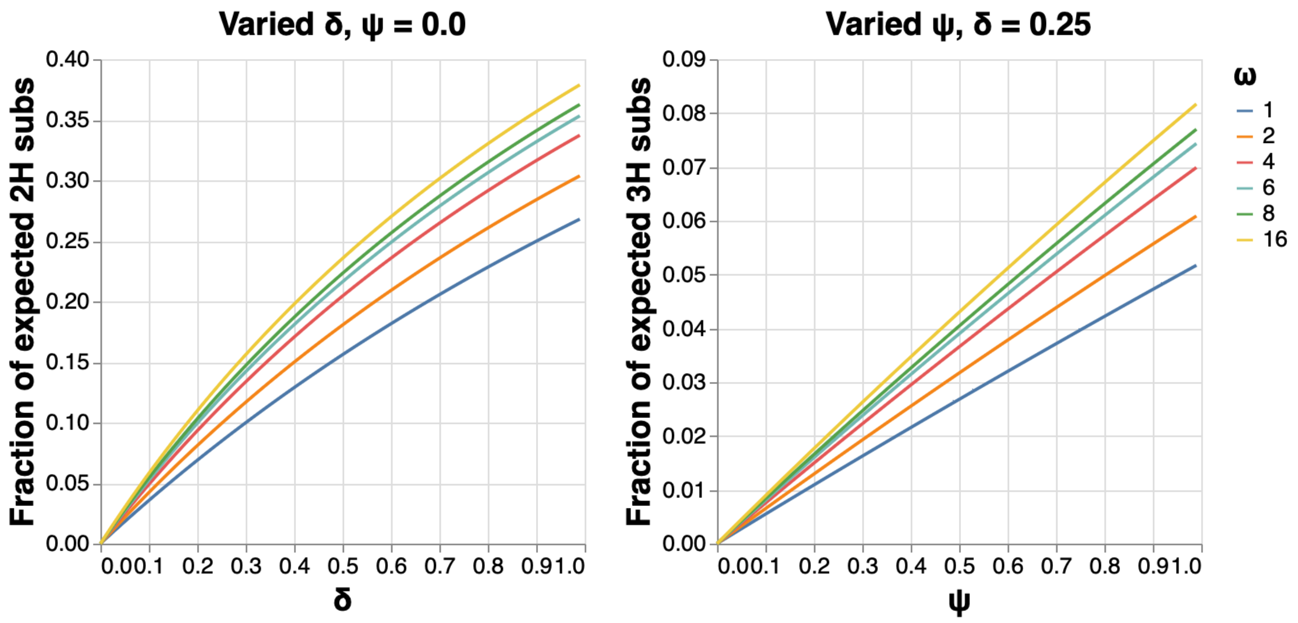

FIG. S3. Fractions of expected substitutions attributable to 2H or 3H substitutions model parameter values used in the 4-taxon simulations. ω refers to the value of the $\omega_3 \geq 1$ parameter.

· doi:10.1093/molbev/mst012 MBE

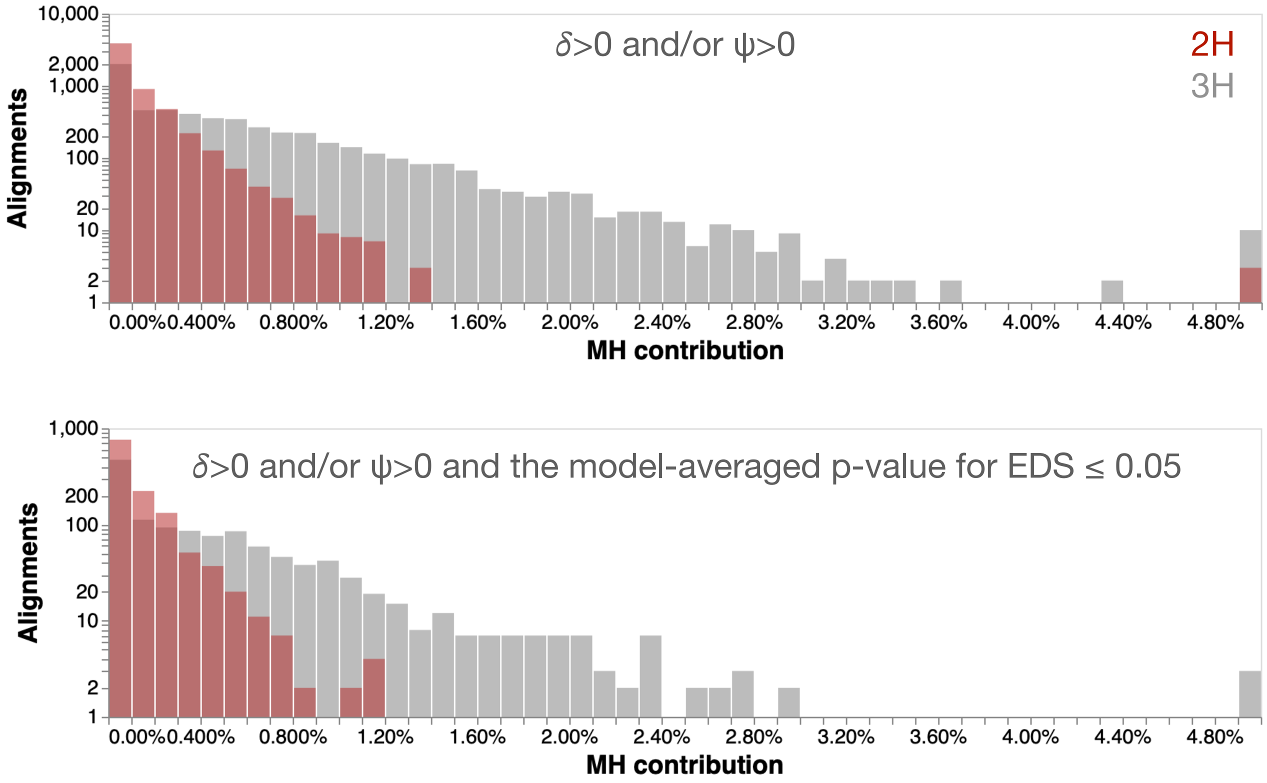

FIG. S4. Fractions of expected substitutions attributable to 2H or 3H inferred from the ? dataset . The top panel shows histograms over alignments with non-zero estimated δ or ψ rates under the +S+MH model. The bottom panel further subsets these to alignments where EDS was detected with the model-averaging approach at $p \leq 0.05$. Both plots are right-censored at 5%, and all the alignments with estimates above this point are grouped in the rightmost bin. Note the logarithmic scale on the y-axis.

Scenario	$\omega_3(p_3)$			δ		ψ		Detection (# if AIC_c is better)			+S+MH pref
	Truth	+S	+S+MH	Truth	+S+MH	Truth	+S+MH	+S	+S+MH	Averaged	by LRT
**Null simulations (no positive selection, MH present)**											
adh/N1	**1.0**(2.38%)	1.00 − 2.42 (1.6%)	1.00 − 2.63 (1%)	0.003	0.00 − 0.04	0.0	0.00 − 0.00	0.03 (97)	0.01 (3)	0.01	0
adh/N2	**1.0**(2.38%)	1.00 − 3.60 (1.7%)	1.00 − 1.93 (0.98%)	0.1	0.00 − 0.12	0.0	0.00 − 0.07	0.1 (80)	0 (20)	0.04	0.14
adh/N3	**1.0**(2.38%)	1.30 − 5.21 (2.3%)	1.00 − 2.26 (1.2%)	0.25	0.10 − 0.27	0.0	0.00 − 0.08	0.26 (47)	0.03 (53)	0.05	0.39
adh/N4	**1.0**(2.38%)	2.07 − 7.98 (2.3%)	1.00 − 2.69 (1.3%)	0.5	0.33 − 0.56	0.0	0.00 − 0.06	0.61 (6)	0.02 (94)	0.02	0.9
adh/N5	**1.0**(2.38%)	3.05 − 19.29 (2.1%)	1.00 − 2.32 (1.1%)	0.75	0.57 − 0.79	0.0	0.00 − 0.12	0.87 (0)	0.03 (100)	0.03	1
Hepatitis D Ag/N1	**1.0**(1.71%)	1.19 − 10.93 (13%)	1.00 − 1.04 (6.9%)	0.14	0.08 − 0.15	0.0	0.00 − 0.00	0.34 (32)	0.01 (68)	0.06	0.54
HIV *vif*/N1	**1.0**(1.00%)	1.07 − 50.21 (14%)	1.00 − 1.90 (7.2%)	0.004	0.00 − 0.00	0.16	0.08 − 0.22	0.32 (32)	0.01 (68)	0.01	0.63
Rhodopsin/N1	**1.0**(0.37%)	3.37 − 14.11 (1.1%)	1.00 − 1.69 (0.6%)	0.35	0.27 − 0.37	0.52	0.27 − 0.64	0.9 (1)	0.02 (100)	0.03	0.99
Strep. PTS/N1	**1.0**(1.56%)	1.48 − 12.41 (1.6%)	1.00 − 5.59 (0.93%)	0.31	0.15 − 0.40	1.1	0.70 − 1.46	0.31 (1)	0.03 (99)	0.03	0.99
**Power simulations (positive selection, MH absent)**											
adh/P1	4.14(2.50%)	3.21 − 4.79 (2.9%)	3.30 − 4.91 (2.8%)	0.0	0.00 − 0.00	0.0	0.00 − 0.00	0.91 (99)	0.85 (1)	0.92	0.01
β-globin/P1	8.925(3.70%)	5.53 − 10.96 (5%)	5.73 − 9.67 (4.8%)	0.0	0.00 − 0.00	0.0	0.00 − 0.03	1 (91)	0.98 (9)	1	0.05
HIV *vif*/P1	2103(0.05%)	1.09 − 3142.84 (9.7%)	1.00 − 2.54 (6.2%)	0.0	0.00 − 0.02	0.0	0.00 − 0.11	0.56 (80)	0.1 (20)	0.53	0.14
Mam. mtDNA/P1	1.434(1.33%)	1.29 − 1.63 (1.3%)	1.21 − 1.49 (1.4%)	0.0	0.00 − 0.03	0.0	0.00 − 0.05	0.65 (90)	0.5 (10)	0.64	0.06
Rhodopsin/P1	6.376(1.31%)	5.13 − 7.38 (1.4%)	5.16 − 7.50 (1.4%)	0.0	0.00 − 0.00	0.0	0.00 − 0.10	1 (99)	0.98 (2)	0.99	0.01
**Power simulations (positive selection, MH present)**											
adh/P2	4.05(2.38%)	3.23 − 5.40 (2.6%)	3.04 − 5.05 (2.5%)	0.003	0.00 − 0.03	0.0	0.00 − 0.06	0.9 (94)	0.75 (6)	0.86	0.02
β-globin/P2	2.834(6.08%)	2.71 − 6.40 (7%)	1.88 − 4.54 (7.4%)	0.24	0.01 − 0.28	0.19	0.00 − 0.14	0.88 (56)	0.4 (44)	0.55	0.33
Hepatitis D Ag/P1	11.3(1.71%)	8.93 − 19.65 (3.7%)	2.40 − 9.86 (9.4%)	0.14	0.07 − 0.17	0.0	0.00 − 0.07	1 (32)	0.58 (68)	0.65	0.57
HIV *vif*/P2	1.226(1.00%)	1.64 − 319.54 (9.4%)	1.00 − 3.12 (6.2%)	0.004	0.00 − 0.00	0.16	0.08 − 0.23	0.46 (35)	0.01 (65)	0.04	0.52
Rhodopsin/P2	5.453(0.37%)	5.61 − 15.94 (0.96%)	1.00 − 6.27 (0.73%)	0.35	0.26 − 0.35	0.52	0.25 − 0.81	1 (1)	0.24 (100)	0.25	0.99
Rhodopsin/P3	5.453(0.37%)	4.14 − 9.24 (1.1%)	1.00 − 5.47 (0.61%)	0.35	0.26 − 0.37	**0.0**	0.00 − 0.18	0.96 (0)	0.22 (100)	0.22	0.97
Rhodopsin/P4	5.453(0.37%)	3.59 − 8.65 (0.72%)	2.23 − 7.11 (0.71%)	**0.10**	0.03 − 0.13	**0.0**	0.00 − 0.08	0.87 (70)	0.32 (30)	0.55	0.18
Rhodopsin/P5	5.453(0.37%)	3.15 − 8.12 (1.1%)	1.37 − 6.64 (0.83%)	**0.20**	0.14 − 0.24	**0.0**	0.00 − 0.09	0.85 (21)	0.27 (79)	0.37	0.7
Rhodopsin/P6	5.453(0.37%)	4.47 − 20.37 (0.6%)	1.62 − 6.06 (0.71%)	**0.00**	0.00 − 0.01	0.52	0.35 − 0.60	0.96 (26)	0.28 (74)	0.39	0.62
Rhodopsin/P7	5.453(**2.1**%)	7.21 − 11.00 (2.2%)	4.65 − 6.46 (2.2%)	0.35	0.28 − 0.38	**0.0**	0.00 − 0.12	1 (3)	0.98 (97)	0.99	0.97
Rhodopsin/P8	5.453(**4.2**%)	7.51 − 9.64 (4.1%)	4.78 − 6.15 (4.4%)	0.35	0.26 − 0.36	**0.0**	0.00 − 0.06	1 (3)	0.99 (97)	1	0.97
Strep. PTS/P1	9.489(1.56%)	17.10 − 71.54 (1.4%)	7.55 − 12.88 (1.6%)	0.31	0.15 − 0.43	1.1	0.65 − 1.45	0.99 (0)	0.97 (100)	0.97	1
SARS-CoV-2 S/P1	5.990(20.12%)	4.64 − 7.63 (27%)	4.86 − 8.94 (22%)	0.012	0.00 − 0.00	0.0	0.00 − 0.00	0.99 (100)	0.97 (0)	0.99	0

Table S1. BUSTED test performance on synthetic data, under model fits from benchmark datasets to parametrize various simulation scenarios simulations (100 replicates each). `Truth` – values used for data generation; parameters changed from their MLE values from the corresponding empirical dataset are shown in **boldface**. For model rate estimates, interquartile range is shown. For proportion estimates, mean value is shown. `Detection` columns shows the fraction of replicates where the LRT for episodic diversifying selection yields $p \leq 0.05$; and the value in parentheses – the number of replicates where the corresponding model was preferred by AIC_c. `Detection / Averaged` – the fraction of replicates where model-averaged LRT p-value was ≤ 0.05. The last column shows the fraction of replicates for which the +S+MH model was preferred to the +S model, using the χ^2_2 based LRT $p \leq 0.05$.

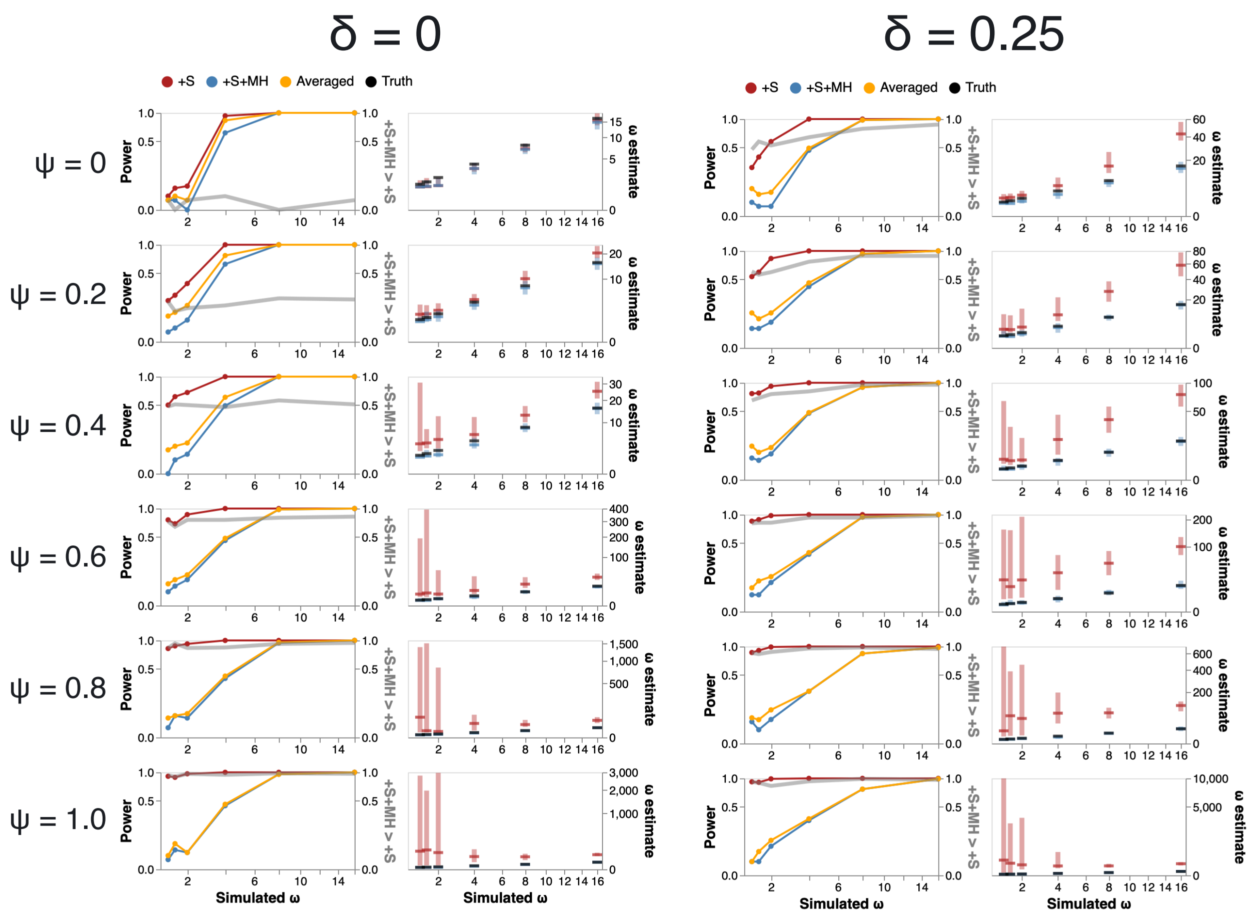

FIG. S5. Model performance on data simulated with EDS when ψ is varied. Left column : detection rate for EDS (at $p \leq 0.05$) as a function of rate ω_3 (effect size) and δ (confounding parameter), and the rate at which +S+MH is preferred to +S by a nested LRT test. Right column: ω_3 estimates (median, IQR) for various simulation scenarios.

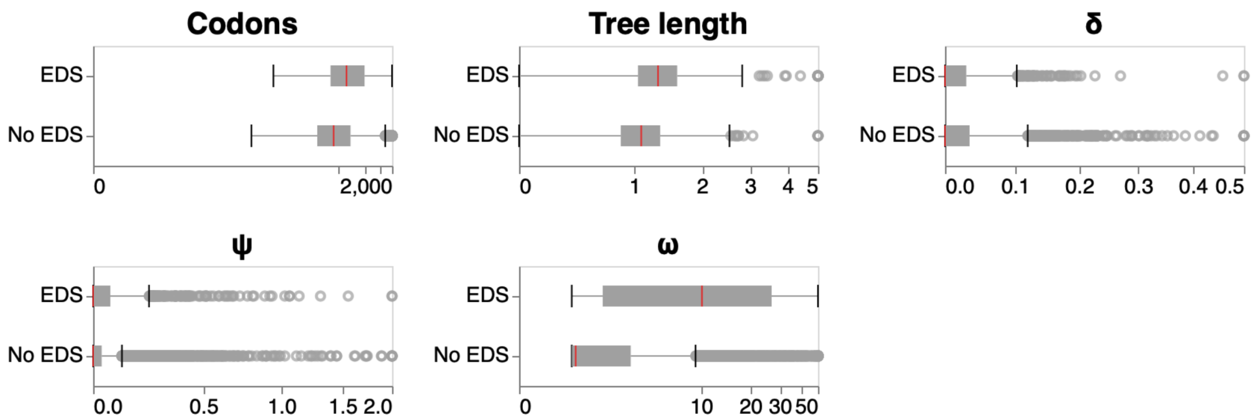

FIG. S6. Correlates of EDS detection. Boxplots (medians are shown in red, and right truncated to improve presentation, i.e. all values greater than the maximum value displayed are censored at that value) of various Enard collection alignment attributes stratified by whether or not EDS was detected using model averaging.

· doi:10.1093/molbev/mst012 MBE

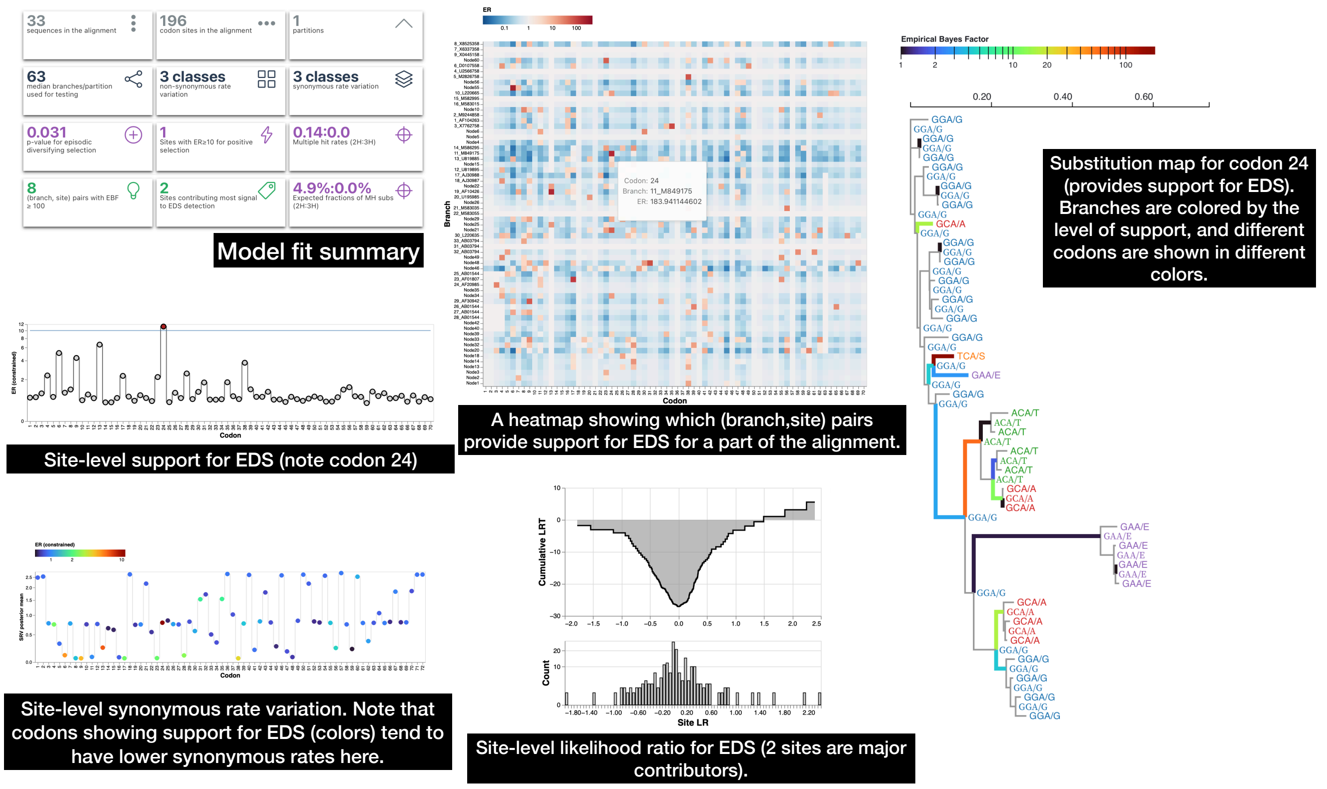

FIG. S7. Data visualization modules . Examples of data visualizations of the +S+MH fit on the Hepatitis D Ag alignment viewable at `vision.hyphy.org/busted` or `https://observablehq.com/@spond/busted`.

Alignment	Branch, site pairs with $EBF \geq 100$				# of sites providing >80% of LRT support				Type
	BUSTED	+S	+MH	+S+MH	BUSTED	+S	+MH	+S+MH	
Sperm lysin	55	35	73	35	23	14	13	8	All, yes
IAV H1N1 HA	21	364	21	330	8	6	6	6	All, yes
rbcL	1335	901	1217	818	30	38	21	9	All, yes
SARS-CoV-2 S	324	167	6	0	5	5	6	6	All, yes
HIV rt	758	0	583	226	10	3	11	2	All, yes
Camelid VHH	175	90	44	8	20	12	9	4	All, yes
Drosophila *adh*	49	38	31	33	4	4	4	3	All, yes
Hepatitis D Ag	37	29	9	8	13	7	5	2	All, yes
Streptococcus PTS	16	53	0	49	8	22	N/A	17	+S/+S+MH, yes
Mam. $\beta-$globin	21	25	9	4	6	5	3	N/A	Discordant
Mammalian mtDNA	588	227	641	165	9	7	8	N/A	Discordant
Rhodopsin	97	80	73	35	14	5	9	N/A	Discordant
HIV *vif*	13	11	0	0	3	1	2	N/A	Discordant

Table S2. Breadth of support for EDS on empirical datasets. For each model, we report the number of (branch,site) pairs where empirical Bayes factor (EBF) support for EDS is >100, and the number of sites whose individual likelihood ratios combine to yield >80% of the total test statistic for the alignment (as shown in Figure S2 for some example datasets

· doi:10.1093/molbev/mst012 MBE

Type	N	# of sites for $>80\%$ of LRT support				Branch, site pairs with $EBF \geq 100$				Fraction with single site signal			
		.	+S	+MH	+S+MH	.	+S	+MH	+S+MH	.	+S	+MH	+S+MH
BUSTED	2805	3	2	3	3	33	25	13	14	11.7%	14.8%	7.3%	7.8%
+S	2607	3	2	3	3	31	29	8	16	12.1%	17.3%	7.1%	8.3%
Best	2425	3	2	3	3	30	28	10	17	12.8%	17.8%	7.3%	8.8%
+S, Best	2411	3	2	3	3	30	28	10	17	12.7%	17.8%	7.2%	8.3%
BUSTED, +S	1957	3	3	3	3	36	30	14	19	11.3%	14.0%	6.9%	7.1%
Averaged	1908	3	3	3	3	32	29	16	22	11.7%	14.8%	7.3%	8.6%
Best, Averaged	1901	3	3	3	3	32	29	16	22	11.8%	14.8%	7.3%	8.6%
+S, Averaged	1898	3	3	3	3	32	30	16	22	11.6%	14.7%	7.3%	8.3%
+S, Best, Averaged	1894	3	3	3	3	32	30	16	22	11.7%	14.7%	7.4%	8.3%
BUSTED, Best	1820	3	3	3	3	35	29	17	20	12.1%	14.6%	7.1%	7.5%
BUSTED, +S, Best	1809	3	3	3	3	35	29	17	20	12.1%	14.5%	7.0%	7.2%
BUSTED, Averaged	1504	3	3	3	3	36	31	20	23	11.2%	12.5%	7.1%	7.4%
BUSTED, Best, Averaged	1499	3	3	3	3	36	31	20	23	11.2%	12.4%	7.1%	7.5%
BUSTED, +S, Averaged	1498	3	3	3	3	36	31	20	23	11.1%	12.3%	7.1%	7.2%
BUSTED, +S, Best, Averaged	1494	3	3	3	3	36	31	20	23	11.1%	12.4%	7.2%	7.2%
+S+MH	984	3	3	3	3	36	32	26.5	30.5	6.6%	6.3%	7.3%	7.5%
Best, +S+MH	976	3	3	3	3	36	32	27	31	6.5%	6.1%	7.2%	7.4%
Averaged, +S+MH	976	3	3	3	3	36	32	27	31	6.5%	6.2%	7.0%	7.2%
Best, Averaged, +S+MH	973	3	3	3	3	36	32	27	31	6.5%	6.1%	7.0%	7.2%
+S, +S+MH	967	3	3	3	3	36	32	27	31	6.3%	6.0%	7.0%	6.8%
+S, Best, +S+MH	966	3	3	3	3	36	32	27	31	6.3%	6.0%	7.0%	6.8%
BUSTED, +S+MH	846	3	3	3	3	39	33	29	32	6.4%	5.7%	7.2%	6.5%
BUSTED, Best, +S+MH	843	3	3	3	3	39	33	29	32	6.3%	5.5%	7.1%	6.3%
BUSTED, Averaged, +S+MH	842	3	3	3	3	39	33	29	32	6.3%	5.6%	6.9%	6.2%
BUSTED, Best, Averaged, +S+MH	841	3	3	3	3	39	33	29	32	6.3%	5.5%	6.9%	6.2%
BUSTED, +S, +S+MH	836	3	3	3	3	39	33	29	32	6.1%	5.4%	6.9%	5.9%
+MH	826	3	3	3	3	35.5	27	33.5	24	5.1%	7.2%	6.7%	5.4%
BUSTED, +MH	819	3	3	3	3	36	27	33	24	5.1%	7.1%	6.3%	5.4%
+S, +MH	649	4	3	3	3	38	31	35	27	4.9%	6.9%	6.0%	4.9%
BUSTED, +S, +MH	645	4	3	3	3	38	31	35	27	5.0%	6.8%	5.9%	4.9%
Best, +MH	644	4	3	3	3	37.5	31	35	27	5.0%	7.0%	6.2%	5.0%
+S, Best, +MH	641	4	3	3	3	38	31	35	27	5.0%	7.0%	6.1%	4.9%
BUSTED, Best, +MH	640	4	3	3	3	38	31	35	27.5	5.0%	6.9%	6.1%	5.0%
BUSTED, +S, Best, +MH	637	4	3	3	3	38	31	35	28	5.0%	6.9%	6.0%	4.9%
Averaged, +MH	627	4	3	3	3	38	32	35	28	5.0%	7.0%	6.1%	4.8%
+S, Averaged, +MH	624	4	3	3	3	38	32	35	28	5.0%	6.9%	6.1%	4.9%
Best, Averaged, +MH	624	4	3	3	3	38	32	35	28	5.0%	6.9%	6.1%	4.8%
BUSTED, Averaged, +MH	623	4	3	3	3	38	32	35	28	5.0%	6.9%	5.9%	4.8%
+S, Best, Averaged, +MH	622	4	3	3	3	38	32	35	28	5.0%	6.9%	6.1%	4.9%
BUSTED, +S, Averaged, +MH	620	4	3	3	3	38	32	35.5	28	5.0%	6.8%	6.0%	4.9%
BUSTED, Best, Averaged, +MH	620	4	3	3	3	38	32	35.5	28	5.0%	6.8%	6.0%	4.9%
BUSTED, +S, Best, Averaged, +MH	618	4	3	3	3	38	32	35.5	28	5.0%	6.8%	6.0%	4.9%
+S+MH, +MH	522	4	3	3	3	38.5	32	36.5	31	5.0%	4.8%	6.1%	5.0%
BUSTED, +S+MH, +MH	520	4	3	3	3	38.5	32	36.5	31	5.0%	4.8%	6.2%	5.0%
Best, +S+MH, +MH	520	4	3	3	3	39	32	37	31	4.8%	4.6%	6.0%	4.8%
Averaged, +S+MH, +MH	520	4	3	3	3	39	32	37	31	4.8%	4.8%	5.8%	4.6%
Best, Averaged, +S+MH, +MH	519	4	3	3	3	39	32	37	31	4.8%	4.6%	5.8%	4.6%
BUSTED, Best, +S+MH, +MH	518	4	3	3	3	39	32	37	31	4.8%	4.7%	6.0%	4.8%
BUSTED, Averaged, +S+MH, +MH	518	4	3	3	3	39	32	37	31	4.8%	4.8%	5.8%	4.6%
All but +S	517	4	3	3	3	39	32	37	31	4.8%	4.7%	5.8%	4.6%
All but BUSTED	517	4	3	3	3	39	32	37	31	4.8%	4.6%	5.8%	4.6%
All	515	4	3	3	3	39	32	37	31	4.9%	4.7%	5.8%	4.7%

Table S3. Breadth of support for EDS on the Enard et al. For each model (. = BUSTED) and groups of alignments based on the EDS detection pattern (see text), we report: (branch,site) pairs where empirical Bayes factor (EBF) support for EDS is >100, the number of sites whose individual likelihood ratios combine to yield $>80\%$ of the total test statistic for the alignment (as shown in Figure S2 for some example datasets, and the fraction of alignments where EDS was detected where a single site provided most of the EDS support .
